# Blood Urea Nitrogen for Short-Term Prognosis in Patients with Cardiogenic Shock Complicating Acute Myocardial Infarction

**DOI:** 10.1155/2022/9396088

**Published:** 2022-03-15

**Authors:** Yuansong Zhu, Bryan Richard Sasmita, Xiankang Hu, Yuzhou Xue, Hongbo Gan, Zhenxian Xiang, Yi Jiang, Bi Huang, Suxin Luo

**Affiliations:** Department of Cardiology, The First Affiliated Hospital of Chongqing Medical University, Chongqing, China

## Abstract

**Purpose:**

Cardiogenic shock (CS) is the leading cause of death in patients with acute myocardial infarction (AMI). Our study aimed to evaluate the short-term prognostic value of admission blood urea nitrogen (BUN) in patients with CS complicating AMI.

**Materials and Methods:**

218 consecutive patients with CS after AMI were enrolled. The primary endpoint was 30-day mortality. The association of admission BUN and 30-day mortality and major adverse cardiovascular event (MACE) was investigated by Cox regression. The integrated discrimination improvement (IDI) and net reclassification improvement (NRI) further examined the predictive value of BUN.

**Results:**

During a period of 30-day follow-up, 105 deaths occurred. Compared to survivors, nonsurvivors had significantly higher admission BUN (*p* < 0.001), creatinine (*p* < 0.001), BUN/creatinine (*p* = 0.03), and a lower glomerular filtration rate (*p* < 0.001). The area under the curve (AUC) of the 4 indices for predicting 30-day mortality was 0.781, 0.734, 0.588, and 0.773, respectively. When compared to traditional markers associated with CS, the AUC for predicting 30-day mortality of BUN, lactate, and left ventricular ejection fraction were 0.781, 0.776, and 0.701, respectively. The optimal cut-off value of BUN for predicting 30-day mortality was 8.95 mmol/L with Youden-Index analysis. Multivariate Cox analysis indicated BUN >8.95 mmol/L was an important independent predictor for 30-day mortality (HR 2.08, 95%CI 1.28–3.36, *p* = 0.003) and 30-day MACE (HR 1.85, 95%CI 1.29–2.66, *p* = 0.001). IDI (0.053, *p* = 0.005) and NRI (0.135, *p* = 0.010) showed an improvement in the accuracy for mortality prediction of the new model when BUN was included compared with the standard model of predictors in previous scores.

**Conclusion:**

An admission BUN >8.95 mmol/L was robustly associated with increased short-term mortality and MACE in patients with CS after AMI. The prognostic value of BUN was superior to other renal markers and comparable to traditional markers. This easily accessible index might be promising for early risk stratification in CS patients following AMI.

## 1. Introduction

Acute myocardial infarction (AMI) complicated by cardiogenic shock (CS) is a high-acuity and complex state of end-organ hypoperfusion that is frequently associated with multisystem organ failure. Despite advances in therapeutic options in recent years, mortality remains high [1, 2]. To assist the triage of patients for specific therapies and to determine prognosis, many investigators have evaluated the predictors of mortality in CS patients. However, there was a wide heterogeneity among these studies, and not all important variables were analyzed [3].

The markers of renal function are known to be closely related to the outcomes in cardiovascular disorders [4, 5]. Routine parameters used for evaluating patients' renal function usually include blood urea nitrogen (BUN), creatinine, glomerular filtration rate (GFR), and sometimes the ratio of BUN and creatinine [6]. While both BUN and creatinine are filtrated through the glomerulus and can reflect GFR, only BUN is reabsorbed from the tubules [6, 7]. In heart failure patients, it was found that elevated BUN was not only a result of damaged renal function but also related to enhanced urea reabsorption because of neurohumoral activation [8]. In fact, BUN has been shown to be an independent predictor of postdischarge all-cause mortality in elderly patients with acute decompensated heart failure, and its prognostic performance was similar to that of B-type natriuretic peptide (BNP) [9].

CS is regarded as the most severe heart failure status after AMI, whereas the association between BUN and the outcome in patients with CS after AMI has not been well-studied. Accordingly, the present study aimed to evaluate the admission BUN in CS complicating AMI for prognostic relevance and to compare its predictive value with renal biomarkers and other well-acknowledged predictors in this population.

## 2. Materials and Methods

It is a retrospective study that is aimed to evaluate the short-term outcomes in patients who developed CS after AMI. The data of consecutive patients diagnosed with AMI in our hospital between January 2013 and September 2020 were collected from the computerized patient record system. Then, patients whose clinical presentation and laboratory examination were in accord with CS were further screened according to the definition of CS [10]. Finally, a total of 245 consecutive patients diagnosed with AMI complicated by CS were identified. AMI was diagnosed when there is a rise and/or fall of cardiac troponin values with at least 1 value above the 99^th^ percentile upper reference limit and at least 1 of the following: symptoms of myocardial ischemia, new ischemic electrocardiogram (ECG) changes, the development of pathological Q waves, imaging evidence of a new loss of viable myocardium, or new regional wall motion abnormality in a pattern consistent with ischemic etiology [11]. CS was defined as the systolic blood pressure (SBP) <90 mmHg under pharmacological and/or mechanical support to maintain SBP ≥90 mmHg, the evidence of end-organ hypoperfusion, such as urine output <30 ml/h, cool extremities, altered mental status, and/or serum lactate >2.0 mmol/L [10]. This study was performed in accordance with the principle of the Declaration of Helsinki, and the research protocol was approved by the institutional ethical review board of The First Affiliated Hospital of Chongqing Medical University (No. 2020-233).

Baseline, laboratory, and procedural data were all extracted from the electronic medical system by experienced doctors and nurses. Baseline characteristics, including age, gender, body mass index, medical histories, clinical presentations, and vital signs, were recorded on admission. Laboratory data, including arterial blood gas, BUN, serum creatinine, cardiac enzymes, and BNP, were also obtained on admission. The reference range of our laboratory for BUN and serum creatinine was 3.6–9.5 mmol/L and 57–111 *μ*mol/L, respectively. GFR was calculated by the CKD-EPI equation, an equation that was proposed in 2009, which is now regarded as one of the most recommended equations for estimated GFR [12]. After admission, patients were given acute coronary artery angiography unless contraindication existed or refused by patients, and the characteristics of the coronary artery were collected. The culprit vessels were treated according to the recommendations for the management of CS complicating AMI [13]. After the intervention procedure, patients were sent to the coronary care unit for electrocardiogram monitoring and further management. Vasoactive agents, antiplatelet drugs, statins, *β*-blockers, angiotensin-converting enzyme inhibitor (ACEI)/angiotensin-converting receptor blocker (ARB), proton pump inhibitor, and other medications were administered according to the guidelines [2, 14].

The primary endpoint of the study was all-cause mortality during the 30-day follow-up. The second endpoint was major adverse cardiovascular events (MACE) that included all-cause mortality, ventricular tachycardia or fibrillation (VT/VF), high-grade atrioventricular block (AVB), and nonfatal stroke during the 30-day follow-up.

The analysis of normality was performed for continuous variables with Kolmogorov–Smirnov test. Continuous variables that were normally distributed were expressed as mean ± standard deviation (SD) and compared with *t*-test, while non-normally-distributed data were expressed as median with interquartile range (IQR) and compared with Mann–Whitney U test. Categorical variables were presented as frequency (percentage) and compared using the Pearson chi-square test. To identify the predictive value of BUN, creatinine, BUN-to-creatinine ratio, and GFR, the receiver operating characteristic curve (ROC) was adopted, and the area under the curve (AUC) for 30-day mortality and 30-day MACE were calculated. The ROC of BUN was also compared to arterial lactate and left ventricular ejection fraction (LVEF). Patients were then divided into 2 groups according to the cut-off value of BUN determined by the Youden-index. The primary and secondary outcomes were compared between the 2 groups. Then, Kaplan–Meier analysis by quartiles and cut-off of BUN was employed to compare all-cause mortality and to plot time-to-event curves, and the comparisons of the curves were achieved by log-rank test. To evaluate and to adjust for confounding factors for 30-day mortality and MACE, a multivariate Cox proportional model was built by forward stepwise variable selection by including parameters with significant *p* values in the univariate analysis. In the Cox regression model, the continuous laboratory variables, including arterial lactate, troponin I (TNI), BNP, GFR, and LVEF, were all converted to categorical variables by their respective Yonden-indexes. The adjusted hazard ratios (HR) with 95% confidence intervals (CIs) were calculated. Specifically, both HRs of BUN as a continuous variable and as a categorical variable were calculated, but not at the same time. The multivariate model only included BUN as a categorical variable for analysis. Later, a standard model for 30-day mortality prediction was developed by including age, SBP, anterior myocardial infarction, lactate, and creatinine that were components of previous scores [3, 15, 16]. Then, the integrated discrimination improvement (IDI) and net reclassification improvement (NRI) were calculated to evaluate the improvement of a predictive value of a new model by replacing creatinine with BUN. Specifically, the category NRI was calculated using 20% and 40% as the thresholds to define the risk grade of 30-day mortality: low risk (<20%), intermediate risk (20–40%), and high risk (>40%), based on a previous risk stratification of CS from the IABP-SHOCK II trial [16]. All statistical analyses were carried out using the SPSS software, version 26.0 (IBM Corp. Armonk, NY, USA) or R, version 3.6.3 (R Foundation for Statistical Computing, Vienna, Austria). Statistical significance was defined as a two-sided *p* value <0.05.

## 3. Results

### 3.1. Baseline Characteristics and Medications

A total of 245 patients were diagnosed with CS complicating AMI, among which 27 patients were excluded because of incomplete data or without undergoing coronary artery angiography, and the remaining 218 patients were included in this study. They were initially divided into 2 groups according to their survival status at the end of 30-day follow-up. During a period of 30-day follow-up, 105 patients died. The baseline characteristics and medications during hospitalization are listed in Table 1. Compared to the survivors, nonsurvivors were older (*p* < 0.001), slightly thinner (*p* = 0.02), more likely to be female (*p* = 0.04) or nonsmokers (*p* < 0.001), and presented with lower SBP (*p* = 0.02). The history of hypertension, diabetes mellitus, dyslipidemia, stroke, and atrial fibrillation were similar between the 2 groups. As for AMI localization, there was no difference between the 2 groups. In terms of medications, the usages of ticagrelor (*p* = 0.02), statins (*p* = 0.001), ACEI/ARB (*p* < 0.001), and *β*-blocker (*p* < 0.001) were higher in survivors, while the usage of dopamine (*p* = 0.007) and norepinephrine (*p* = 0.003) was higher in nonsurvivors.

### 3.2. Laboratory, Echocardiographic, and Angiographic Findings

**Figure 1:**
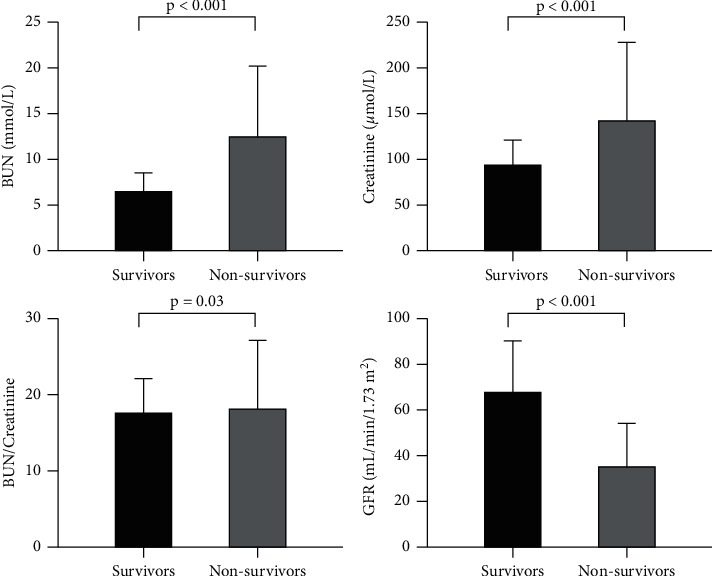
The admission levels of BUN, creatinine, BUN-to-creatinine ratio, and GFR of survivors and nonsurvivors. Data were shown in median with IQR.

The laboratory, echocardiographic, and angiographic findings of all patients are summarized in Table 2. Lactate, BNP, D-dimer, and white blood cell in nonsurvivors were significantly higher than in survivors (all *p* < 0.05). Nonsurvivors also had lower left ventricular ejection fraction (LVEF) (*p* < 0.001), larger left ventricular end-diastolic dimension (*p* < 0.001), and more septal perforation occurrence (*p* < 0.001).  Figure 1 compares the median with the IQR of BUN, creatinine, BUN-to-creatinine ratio, and GFR of the two groups. Compared with the survivors, nonsurvivors had significantly elevated admission BUN (*p* < 0.001), creatinine (*p* < 0.001), BUN-to-creatinine ratio (*p* = 0.03), and reduced GFR (*p* < 0.001).

### 3.3. Association of BUN with the Outcomes

**Figure 2:**
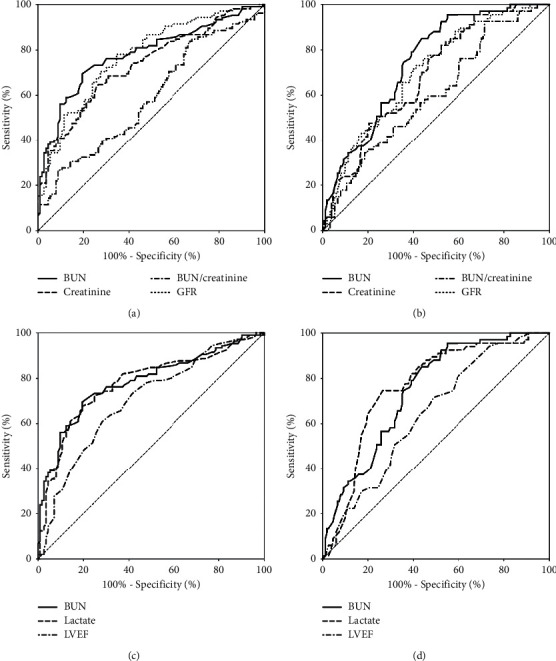
(a) The ROC of BUN, creatinine, BUN-to-creatinine ratio, and GFR for 30-day mortality. The AUC were 0.781, 0.734, 0.588, and 0.773, respectively. (b) The ROC of BUN, creatinine, BUN-to-creatinine ratio, and GFR for 30-day MACE prediction, with the AUC of 0.744, 0.687, 0.600, and 0.702. (c) The ROC of BUN, lactate, and LVEF for 30-day mortality prediction. The AUC were 0.781, 0.776, and 0.701, respectively. (d) The ROC of BUN, lactate, and LVEF for 30-day MACE prediction, with the AUC of 0.744, 0.760, and 0.640, respectively.

The ROC of BUN, creatinine, BUN-to-creatinine ratio, and GFR for predicting 30-day mortality and 30-day MACE were presented in figures 2(a) and 2(b). The AUC of the 4 indexes for 30-day mortality were 0.781, 0.734, 0.588, and 0.773, respectively. The AUC for 30-day MACE were 0.744, 0.687, 0.600, and 0.702, respectively. BUN had the highest AUC for both 30-day mortality and MACE, followed by GFR. With regard to the already known indicators of organ hypoperfusion, the AUC of arterial lactate and LVEF were 0.776 and 0.701 for 30-day mortality, 0.760 and 0.640 for 30-day MACE, as presented in figures 2(c) and 2(d).

**Figure 3:**
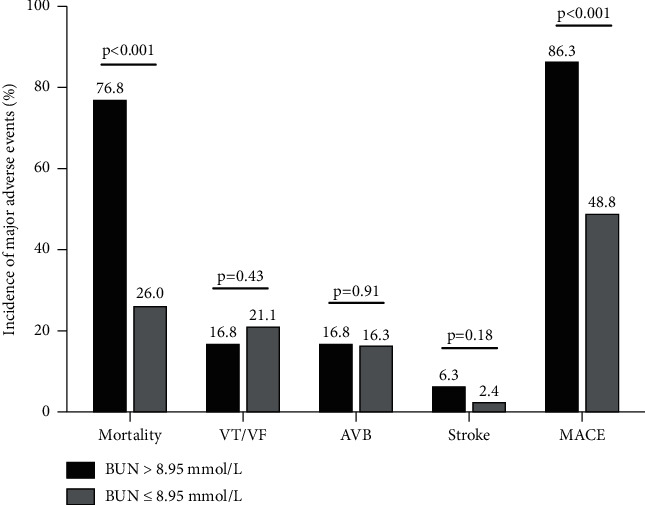
The 30-day outcomes of patients with an admission BUN >8.95 *μ*mol/L and ≤8.95 *μ*mol/L. VT/VF: ventricular fibrillation or ventricular tachycardia, AVB: high-grade atrioventricular block, and MACE: major adverse cardiovascular event.

**Figure 4:**
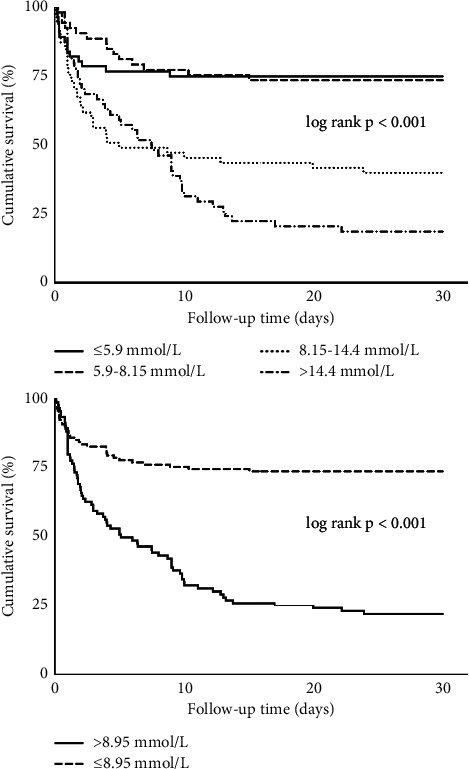
The Kaplan–Meier curves after 30-day all-cause mortality of patients according to quartiles (Quartile 1–4: ≤5.9 mmol/L, 5.9–8.15 mmol/L, 8.15–14.4 *μ*mol/L and >14.4 *μ*mol/L, log rank *p* < 0.001) and cut-off of BUN (≤8.95 mmol/L and >8.95 mmol/L, log rank *p* < 0.001).

The optimal cut-off point for BUN to predict the 30-day mortality in CS patients was determined by Yonden-index with the level of 8.95 mmol/L, with a sensitivity of 0.70, and a specificity of 0.81. The patients were then divided into 2 groups based on the cut-off value, and their outcomes were compared between the 2 groups (Figure 3). The 30-day mortality was significantly higher in the BUN >8.95 mmol/L group (76.8% vs 26.0%, *p* < 0.001). Although the adverse events of VT/VF, AVB, and stroke did not differ between the 2 groups, the total MACE was significantly higher in the group with BUN >8.95 mmol/L (86.3% vs 48.8%, *p* < 0.001). The Kaplan–Meier curves by quartiles (quartile 1: ≤5.9 mmol/L, quartile 2: 5.9–8.15 mmol/L, quartile 3: 8.15–14.4 mmol/L, quartile 4: >14.4 mmol/L) and by cut-off of 8.95 mmol/L for 30-day all-cause mortality were displayed in Figure 4. It was revealed that patients with higher BUN had significantly higher cumulative mortality within 30 days than patients with low BUN (all log-rank *p* < 0.001).

Potential risk factors associated with 30-day mortality and MACE are listed in Table 3. Variables with a *p* value less than 0.05 in the univariate Cox regression analysis were included in the multivariate model. As a continuous variable, BUN was positively associated with an increased risk of 30-day mortality (HR 1.06, 95%CI 1.04–1.08, *p* < 0.001). As a categorical variable, after adjustment, BUN >8.95 mmol/L was identified as an important independent predictor for 30-day mortality (HR 2.08, 95%CI 1.28–3.36, *p* = 0.003). Of note, GFR did not remain significant after adjustment. Other independent predictors for 30-day mortality included age (HR 1.03, 95%CI 1.01–1.05, *p* = 0.009), SBP (HR 0.98, 95% CI 0.97–1.00, *p* = 0.01), lactate >4.2 mmol/L (HR 2.59, 95% CI 1.66–4.04, *p* < 0.001), BNP >400 pg/mL (HR 1.99, 95%CI 1.17–3.37, *p* = 0.01), and LVEF ≤50% (HR 1.73, 95% CI 1.11–2.69, *p* = 0.02). Also, as a continuous variable, BUN was positively associated with an increased risk of 30-day MACE (HR 1.04, 95%CI 1.02–1.06, *p* < 0.001). As a categorical variable, after multivariable adjustment, BUN >8.95 mmol/L remained an independent predictor for 30-day MACE (HR 1.85, 95% CI 1.29–2.66, *p* = 0.001). Only age (HR 1.02, 95%CI 1.00–1.03, *p* = 0.04) and lactate >4.2 mmol/L (HR 2.69, 95%CI 1.89–3.82, *p* < 0.001) were also identified as predictors of 30-day MACE after CS.

**Figure 5:**
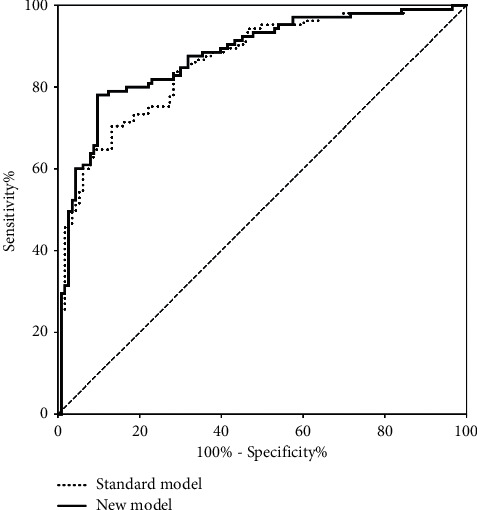
The standard model for 30-day mortality prediction showed an AUC of 0.860, while the AUC of the new model increased to 0.878 when creatinine was replaced by BUN.

The standard model for 30-day mortality prediction showed an AUC of 0.860, while the AUC of the new model increased to 0.878 when creatinine was replaced by BUN, as shown in Figure 5. Moreover, the IDI (0.053, *p* = 0.005) and NRI (0.135, *p* = 0.010) showed an improvement in the accuracy for mortality prediction of the new model when BUN was included, compared with the standard model of predictors in previous scores. The IDI and NRI are listed in Table 4, alongside their 95% CIs.

## 4. Discussion

The major findings of this study are as follows: firstly, in AMI patients who developed CS, admission BUN level was associated with short-term prognosis, and a BUN level higher than 8.95 mmol/L was an independent risk factor for 30-day mortality and 30-day MACE. Secondly, among BUN, creatinine, BUN-to-creatinine ratio, and GFR, BUN is the most effective predictor for short-term prognosis in AMI complicating CS. Thirdly, the prognostic value of BUN was comparable to traditional markers associated with CS. Finally, BUN may effectively add the accuracy of previous scoring systems of short-term mortality in CS. Our present study demonstrated the validity of BUN as a biomarker for prognostic evaluation in CS patients following AMI.

CS is a low-cardiac-output state resulting in life-threatening end-organ hypoperfusion and hypoxia, and AMI with left ventricular dysfunction remains the most common cause of CS [1, 2]. Despite the progress made in the reperfusion therapy, the in-hospital mortality of CS remains high. Therefore, identifying patients that developed CS at the high risk of death as soon as possible is one the most crucial challenges facing the medical staff in the CCU. Although currently several risk scores for CS exist, the sophisticated algorithm and the variability of these scoring systems have limited their utility during practice [16–18].

BUN is a protein metabolic product generated mainly in the liver and excreted through the kidney, and traditionally, the serum BUN level represents the balance between urea production and renal excretion [19]. For decades, BUN has shown its value not only as a marker of renal damage but also a prognostic factor in many clinical conditions. During upper gastrointestinal bleeding, BUN is elevated because of enhanced urea production and can reflect the severity of bleeding [20]. In the general population of critically ill patients in ICU, BUN has demonstrated promise as a practical tool in acute decision making [21, 22]. Such prognostic value of BUN was also seen in acute ischemic stroke [23], acute decompensated or chronic heart failure [9, 24–26], and acute pulmonary embolism [27]. In patients with AMI, the BUN level was also found to be an important predictor of long-term mortality. [28] However, to date, few data are available on the relationship between BUN and the outcomes in patients who developed CS after AMI. Our study confirmed that admission BUN level was independently associated with 30-day mortality and MACE in patients who developed CS after AMI.

The reason why BUN is associated with the short-term outcomes in patients with CS remains to be elucidated. However, several mechanisms may be addressed. In the absence of enhanced production of urea, elevated BUN usually represents a decrease of GFR [6, 19]. In the setting of AMI, especially when CS occurs, multiple factors may contribute to the acute kidney injury (AKI) and result in the decrease of GFR and the increase of BUN. These factors include reduced cardiac output, systemic congestion, the activation of systemic vasoconstriction, the usage of angiotensin-converting enzyme inhibitors or diuretics, and sometimes, the administration of contrast media during revascularization [8, 29]. The elevations of BUN may occur even without the impairment of GFR. As urea reabsorption through proximal and distal tubules is a passive process linked to sodium and water reabsorption, conditions, such as insufficient cardiac output associated with water and sodium reabsorption may also lead to an increase of BUN. Therefore, the elevations of BUN may also represent the appropriate adjustment to the systemic hypoperfusion without substantial detriment to the kidney [7, 9, 24, 25]. CS patients are usually hypovolemic because of the reduced cardiac output, sweating, or sometimes severe vomiting, which could all result in elevated BUN. Therefore, elevated BUN, to some extent, also represents the relatively insufficient blood volume and concentrated blood, which, in turn, may increase the risk of adverse events.

The ROC analysis revealed that among BUN, creatinine, BUN-to-creatinine ratio, and GFR, BUN had the highest efficacy for predicting 30-day mortality and 30-day MACE. The observation was similarly seen in heart failure patients in some recent studies [9, 24–26]. Creatinine is freely filtered at the glomerulus and not reabsorbed, while urea is reabsorbed in both proximal and distal renal tubules. In the collecting duct, the urea reabsorption is flow-dependent so that more urea is reabsorbed as urine flows decrease [28]. Therefore, BUN may better reflect the cumulative hemodynamic effects and neurohormonal changes of severe heart failure than other renal markers in the setting of CS after AMI. Moreover, the predictive value of BUN was comparable to lactate, the widely known marker for organ hypoperfusion and an established predictor for mortality in CS [16, 30]. Such results indicate that the elevations of BUN and lactate reflect similar hemodynamic changes in CS.

After comparison, the optimal cut-off value for BUN to predict 30-day mortality in our study was 8.95 mmol/L, which was a practical value in critical CS patients and comparable to cut-offs identified in some other studies. Aronson et al. [28] proved that a BUN higher than 25 mg/dL (8.93 mmol/L) was an effective predictor for long-term mortality in AMI patients. In ICU, Bernhard Wernly et al. [21] identified 9.7 mmol/L as the optimal cut-off value for long-term mortality prediction in critically ill patients. Furthermore, studies have shown when patients were divided into more groups according to BUN levels in different situations, even subtle changes in BUN were associated with worse outcomes [22–25]. Such association was also seen in our Kaplan–Meier analysis when patients were divided into quartiles.

The NRI is a method that involves classifying patients into risk categories and determines how well a new model reclassifies patients into risk categories compared with the previous model [31]. The IDI calculation is another method to assess reclassification that does not rely on prespecified risk categories but represents a continuous measure [32]. Here, the addition of BUN to the traditional model not only improved the predictive power for 30-day mortality in CS patients (as assessed by AUC of ROC curve) but also the reclassification ability of subjects into different risk categories through NRI and IDI, indicating that BUN may effectively add the accuracy of previous scoring systems.

Our study has some clinical implications. Firstly, because of its convenience, efficiency, and low cost, admission BUN can provide initial risk stratification and prognostic evaluation. Secondly, the predictive value of admission BUN is superior to creatinine and GFR and comparable to some traditional markers, indicating its promising prognostic performance in CS complicating AMI. Putting BUN into the risk stratification model of CS may add the model's accuracy. Finally, we believe monitoring BUN can help optimize management strategies, such as predicting the risk of continuous renal replacement therapy (RRT) because studies have shown elevated baseline BUN was associated with increased risk of AKI in patients with postcardiotomy CS [33]. Recently, Gaudry et al. [34] found in severe AKI patients' BUN concentration higher than 112 mg/dL (39.87 mmol/L) would mandate immediate RRT and longer postponing of RRT initiation did not confer an additional benefit and was associated with potential harm. Therefore, BUN may also play a role in guiding the future management of patients that require RRT.

This study also has some limitations. Firstly, since not all patients received a series of assaying the BUN value, we only analyzed the association of admission BUN level, with the short-term outcome, and dynamic change of BUN may provide better prognostic value. Secondly, BUN value was affected by many factors in addition to the cardiac or renal causes, and some factors were not considered in our present study. Last but not least, some confounders could not be ruled out because of the retrospective and single-center nature of the study, which means more studies are required to further assess the predictive value of BUN in the setting of CS after AMI.

## 5. Conclusions

An admission BUN >8.95 mmol/L was robustly associated with increased short-term mortality and MACE in patients with CS after AMI. The prognostic value of BUN was superior to other renal markers and comparable to traditional prognostic markers. This easily accessible index might be promising for early risk stratification in CS patients following AMI.

## Figures and Tables

**Table 1:** Baseline characteristics and medications of the study population.

Variables	Total (*n* = 218)	CS survivors (*n* = 113)	CS nonsurvivors (*n* = 105)	*p* value
Age (years)	72.5 (63, 78)	66 (59, 75)	75 (69, 81)	<0.001
Male (%)	142 (65.1%)	81 (71.7%)	61 (58.1%)	0.04
BMI (kg/m^2^)	23.1 ± 3.0	23.6 ± 2.9	22.6 ± 3.1	0.02
Hypertension (%)	109 (50%)	57 (50.4%)	52 (49.5%)	0.89
Diabetes (%)	73 (33.5%)	35 (31.0%)	38 (36.2%)	0.42
Dyslipidemia (%)	15 (6.9%)	8 (7.1%)	7 (6.7%)	0.90
Smoking (%)	115 (52.8%)	73 (64.6%)	42 (40.0%)	<0.001
History of stroke (%)	15 (6.9%)	5 (4.4%)	10 (9.5%)	0.14
History of AF (%)	13 (6.0%)	6 (5.3%)	7 (6.7%)	0.67
Prior MI (%)	12 (5.5%)	3 (2.7%)	9 (8.6%)	0.06
SBP (mmHg)	85 (78, 93)	87 (79, 99)	85 (78, 90)	0.02
DBP (mmHg)	56 (50, 63)	57 (51, 64)	55 (48.5, 60.5)	0.17
Heart rate (bpm)	87.6 ± 28.1	84.1 ± 27.8	91.4 ± 28.0	0.06
MI localization (%)				
Anterior	80 (36.7%)	39 (34.5%)	41 (39.0%)	0.49
Inferior	94 (43.1%)	53 (46.9%)	41 (39.0%)	0.24
Lateral	18 (8.3%)	7 (6.2%)	11 (10.5%)	0.25
Right ventricle	27 (12.4%)	18 (15.9%)	9 (8.6%)	0.10
Medications (%)				
Aspirin	201 (92.2%)	106 (93.8%)	95 (90.5%)	0.36
Clopidogrel	114 (52.3%)	61 (54.0%)	53 (50.5%)	0.61
Ticagrelor	115 (52.8%)	68 (60.2%)	47 (44.8%)	0.02
Statins	201 (92.2%)	111 (98.2%)	90 (85.7%)	0.001
ACEI/ARB	74 (33.9%)	53 (46.9%)	21 (20.0%)	<0.001
*β*-blocker	123 (56.4%)	77 (68.1%)	46 (43.8%)	<0.001
Diuretics	119 (54.6%)	61 (54.0%)	58 (55.2%)	0.85
Dopamine	106 (48.6%)	45 (39.8%)	61 (58.1%)	0.007
Norepinephrine	30 (13.8%)	8 (7.1%)	22 (21.0%)	0.003
Nitrates	63 (28.9%)	34 (30.1%)	29 (27.6%)	0.69
Digitalis	28 (12.8%)	14 (12.4%)	14 (13.3%)	0.84

Data are expressed as mean ± SD, median (IQR) or number (percentage). CS: cardiogenic shock, BMI: body mass index, AF: atrial fibrillation, MI: myocardial infarction, SBP: systolic blood pressure, DBP: diastolic blood pressure, and ACEI/ARB: angiotensin-converting enzyme inhibitor or angiotensin receptor blocker.

**Table 2:** Laboratory, echocardiographic, and angiographic findings.

Variables	Total (*n* = 218)	CS survivors (*n* = 113)	CS nonsurvivors (*n* = 105)	*p* value
PO_2_ (mmHg)	84 (63, 110)	88 (64, 110)	79 (60.5, 111)	0.24
PCO_2_ (mmHg)	32 (27, 37)	34 (28, 37.5)	30 (26, 37)	0.02
Lac (mmol/L)	3.4 (1.9, 6.5)	2.3 (1.6, 3.6)	5.9 (3.0, 8.6)	<0.001
TNI (ng/mL)	4.4 (0.9, 10.9)	3.7 (0.7, 9.9)	4.9 (1.3, 16.0)	0.15
BNP >400pg/mL (%)	129 (59.2)	45 (39.8)	84 (80.0)	<0.001
D-dimer (ng/mL)	1.1 (0.6, 2.5)	0.8 (0.3, 1.8)	1.8 (0.9, 3.3)	<0.001
HbA1C (%)	6.3 (5.8, 7.4)	6.3 (5.9, 7.2)	6.3 (5.8, 7.8)	0.68
WBC (*∗* 10^9^/L)	10.5 (7.5, 15.0)	8.2 (6.6, 10.8)	13.3 (10.1, 18.1)	<0.001
Hb (g/L)	120.9 ± 22.6	123.3 ± 19.5	118.4 ± 25.4	0.11
LVEF (%)	48.5 ± 9.8	51.7 ± 9.0	45.0 ± 9.5	<0.001
LVEDD (mm)	52.2 ± 7.1	50.5 ± 5.4	54.0 ± 8.2	<0.001
Mitral regurgitation (%)	93 (42.7)	45 (39.8)	48 (45.7)	0.38
Ventricular aneurysm (%)	54 (24.8)	23 (20.4)	31 (29.5)	0.12
Septal perforation (%)	13 (6.0)	0 (0)	13 (12.4)	<0.001
MVD (%)	91 (41.7)	51 (45.1)	40 (38.1)	0.29

Data are expressed as mean ± SD, median (IQR) or number (percentage). CS: cardiogenic shock, PO_2_: oxygen partial pressure, PCO_2_: carbon dioxide partial pressure, Lac: lactate, TNI: cardiac troponin I, BNP: brain natriuretic peptide, WBC: white blood cell, Hb: hemoglobin, LVEF: left ventricular ejection fraction, LVEDD: left ventricular end-diastolic dimension, and MVD: multivessel disease.

**Table 3:** Predictors for 30-day mortality and 30-day MACE by univariate and multivariate Cox analysis.

Predictors for 30-day mortality	Univariate analysis		Multivariate analysis	
	HR (95%CI)	*p* value	HR (95%CI)	*p* value
Age, per 1-year increase	1.05 (1.03–1.06)	<0.001	1.03 (1.01–1.05)	0.009
Male	0.62 (0.42–0.92)	0.02		
SBP, per mmHg increase	0.98 (0.97–0.99)	0.03	0.98 (0.97–1.00)	0.01
Lac >4.2 mmol/L	4.33 (2.87–6.56)	<0.001	2.59 (1.66–4.04)	<0.001
BNP >400 pg/mL	3.83 (2.37–6.19)	<0.001	1.99 (1.17–3.37)	0.01
BUN (continuous)	1.06 (1.04–1.08)	<0.001		
BUN >8.95 mmol/L	4.09 (2.69–6.22)	<0.001	2.08 (1.28–3.36)	0.003
GFR ≤55 mL/min/1.73 m^2^	3.53 (2.23–5.57)	<0.001		
LVEF ≤50%	2.64 (1.75–4.00)	<0.001	1.73 (1.11–2.69)	0.02
Predictors for 30-day MACE	Univariate analysis		Multivariate analysis	
	HR (95%CI)	*p* value	HR (95%CI)	*p* value
Age, per 1-year increase	1.02 (1.01–1.04)	0.001	1.02 (1.00–1.03)	0.04
Male	0.88 (0.63–1.24)	0.47		
Lac >4.2 mmol/L	3.23 (2.30–4.53)	<0.001	2.69 (1.89–3.82)	<0.001
BNP >400 pg/mL	1.89 (1.35–2.66)	<0.001		
BUN (continuous)	1.04 (1.02–1.06)	<0.001		
BUN >8.95 mmol/L	2.63 (1.88–3.69)	<0.001	1.85 (1.29–2.66)	0.001
GFR ≤55 mL/min/1.73 m^2^	2.17 (1.53–3.08)	<0.001		
LVEF ≤50%	1.80 (1.28–2.51)	0.001		

SBP: systolic blood pressure, Lac: lactate, TNI: cardiac troponin I, BNP: brain natriuretic peptide, BUN: blood urea nitrogen, GFR: glomerular filtration rate, and LVEF: left ventricular ejection fraction.

**Table 4:** Comparison in predicting 30-day mortality between the standard model and the new model.

30-day mortality									
Patients with events					Patients without events				
New model					New model				
Standard	<20%	20–40%	>40%	All	Standard	<20%	20–40%	>40%	All
<20%	4	0	1	5	<20%	47	2	1	50
20–40%	2	12	6	20	20–40%	10	18	4	32
>40%	1	0	79	80	>40%	0	8	23	31
All	7	12	86	105	All	57	28	28	113
			NRI+ = 0.038				NRI- = 0.097		
NRI [95%CI]			0.1354 [0.0323–0.2385]						*p* = 0.010
IDI [95%CI]			0.0526 [0.0158–0.0894]						*p* = 0.005

NRI: net reclassification improvement, IDI: integrated discrimination improvement. The NRI was calculated using 20% and 40% as the thresholds to define the risk grade of 30-day mortality.

## Data Availability

The data used to support the findings of this study are available from the corresponding authors upon request.
